# Intersectional Ageism: Prescriptive Stereotypes and Moral Judgments of Older Adults

**DOI:** 10.1093/geroni/igaf122.917

**Published:** 2025-12-31

**Authors:** Hannah Gans

**Affiliations:** University of Toronto, Toronto, Ontario, Canada

## Abstract

Research on ageism has primarily focused on descriptive stereotypes, while prescriptive expectations—societal beliefs about how older adults should behave—have received less attention, particularly in relation to intersecting identities. When older adults do not conform to these expectations, they may experience negative social consequences, and drawing from intersectionality research, these consequences are likely to vary among older adults with different intersecting identities. To examine these dynamics, we surveyed 355 young adults on societal pressures placed on older adults with intersecting identities. Using a modified Succession, Identity, and Consumption scale (North & Fiske, 2013), we varied the target’s race (Black, White) and gender (man, woman). In a separate analysis, participants also rated the perceived immorality of these targets in society. We found that succession-based prescriptive stereotypes—specifically the expectation that older adults should make room for younger generations—varied by intersectional identity, such that older White men were most strongly expected to adhere to this stereotype. A separate analysis of moral violation ratings revealed that perceptions of immorality also varied across intersectional targets, with older White men rated as the greatest moral violators in society. These findings demonstrate that prescriptive age stereotypes and moral judgments are shaped by intersecting identities. Older White men were most strongly expected to step aside for younger generations and were also rated as the greatest moral violators in society. This highlights the need for further research on how intersectional biases shape perceptions of aging and the distinct social consequences older adults face in different domains.

